# Microplastics pollution in tropical lakes: water, zooplankton, and fish in Central Mexico

**DOI:** 10.1007/s10661-024-12978-4

**Published:** 2024-08-15

**Authors:** Jorge Jiménez-Contreras, Raquel I. Fernández-Medina, Mario A. Fernández-Araiza

**Affiliations:** Laboratorio de Producción Acuícola, Universidad Nacional Autónoma de México, Campus IztacalaLos Reyes Iztacala, CP 54090 Tlalnepantla, Estado de México México

**Keywords:** Anthropogenic stressors, *Chirostoma*, Emerging pollutants, Freshwater systems, Raman

## Abstract

The presence of microplastics in freshwater systems can have harmful effects on the food chain. Zooplankton, especially suspension and filter feeders, can ingest microplastics, which can cause adverse effects and transfer them to higher trophic levels. Here, we analyze the presence, abundance, and distribution of microplastics in surface water, zooplankton, and fish in two tropical lakes in central Mexico. We collected water samples in triplicate at three sites in each lake and 120 fish of the genus *Chirostoma*. From each water sample, 300 rotifers and 150 microcrustaceans were randomly isolated and processed independently. Of the particles found in the water, zooplankton, and fish from both lakes, the fragments were the predominant ones. The total abundance of microplastics in the water column of both lakes varied between 1.2 and 17.0 items L^−1^. In zooplankton, fragments were found predominantly with up to 0.1 items ind^−1^, while in fish, up to 4.5 items ind^−1^ was recorded. Our results confirm the presence of microplastics in different compartments of the food webs of freshwater bodies, water column, zooplankton, and fish. Further work is required on the possible effects of these stressors at the different trophic levels.

## Introduction

Different characteristics of plastic, such as its durability, corrosion resistance, and low cost, generate great social benefits, which is why it has been positioned in practically all aspects of modern life (Andrady & Neal, 2009; Phuong et al., 2016). However, it has also resulted in large quantities of waste that, if improperly managed, means a potential source of contamination of terrestrial, marine, and freshwater environments (Rillig, 2012; Lambert et al., 2013; Eerkes-Medrano et al., 2015). Plastic particles (< 5 mm) are defined as microplastics (MPs) (Thompson et al., 2009), which, depending on their origin, are classified as primary when they are manufactured of microscopic sizes, such as personal care products, and as secondary, formed by the fragmentation of larger plastics by physical or chemical processes (Boucher & Friot, 2017). Due to their small size and low density, microplastics can be easily transported through the air and enter most water bodies (Dris et al., 2016; Rochman, 2018). Microplastics have been widely studied in marine environments (Andrady, 2011; Thompson et al., 2004), where the adverse effects they can cause on marine biota have been revealed (Murray and Cowie, 2011; Cole et al., 2013; Lusher et al., 2013; Nobre et al., 2015). Evidence suggests that microplastic pollution is ubiquitous in terrestrial, marine, estuarine, and freshwater environments (Cole et al., 2011; Forero-López et al., 2021; Rochman, 2018; Xiong et al., 2018). However, the first records in freshwater environments are about 10 years old (Eriksen et al., 2013), so most works have focused on recording microplastic presence, abundance, and distribution in rivers and lakes (reviewed in Zhao et al., 2022).

In freshwater systems, microplastics have been found at average concentrations of 892,777 p/km^2^ in rivers in Europe (Mani et al., 2015), and in lakes in the USA, concentrations of up to 32 particles/m^3^ have been recorded (Baldwin et al., 2016), while in China, between 0.013 and 930 items L^−1^ (Han et al., 2020; Mao et al., 2020) and between 35 and 8925 items/m^3^ (Jian et al., 2020; Wang et al., 2017) have been recorded in rivers and lakes respectively. The size and presence of microplastics in freshwater systems can have harmful effects on the food chain since various groups of zooplankton, especially suspension and filter feeders, can ingest them directly, affecting them and also transferring them to higher trophic levels (Scherer et al., 2018). However, the literature on freshwater systems is limited and still inconclusive (Nelms et al., 2018). It has been proven that some species of rotifers (Drago et al., 2020; Jeong et al., 2016), cladocerans (De Felice et al., 2019; Rehse et al., 2016), and copepods (Cole et al., 2013) can consume microplastics of different sizes and their consumption can have adverse effects on their demographic variables. Thus, at higher levels of the lake food chain, microplastics can be ingested through two routes: direct ingestion (e.g., zooplanktivorous fish) and indirect ingestion through prey (e.g., piscivorous fish) (Scherer et al., 2018).

Reports on the ingestion of microplastics in freshwater fish represent a low percentage (38%) since most of the work has been carried out on species from marine environments (62%); however, species such as *Cyprinus carpio* and *Oreochromis niloticus* have received about a hundred publications (reviewed by Galafassi et al., 2021). Most of the reports on freshwater fish have been carried out mainly in laboratory work (Parker et al., 2021). Field studies (Lin et al., 2020; Park et al., 2020a, 2020b; Garcia et al., 2021) have recorded that the abundance of microplastics in freshwater fish can vary between 0 and 48 particles ind^−1^. The abundance of microplastics in fish is not necessarily related to the abundance of microplastics in the water column (O'Connor et al., 2020; Wang et al., 2020); it may also be related to the concentration of food or even the type and color of microplastics resembling food particles (Kim et al., 2019). In Mexico, work has been carried out with microplastics in the gastrointestinal tract of a fish of high regional consumption (*Oreochromis niloticus*), where they have found abundances of up to 24 items fish^−1^, which may represent a possible trajectory for human exposure (Martinez-Tavera et al., 2021). Also, in the central region of Mexico, there are other species of fish, which, due to their high regional consumption and mode of consumption, may represent a greater risk for human exposure to microplastics, such as pez blanco (*Chirostoma*).

In the central region of Mexico, there are 18 freshwater fish species from the Atherinopsidae family, particularly from the genus *Chirostoma*, commonly known as pez blanco or charales. They are species that originated in this region of the country and, given their restricted distribution, are considered endemic (Barbour, 1973; Berlanga-Robles et al., 2002). Species of this genus have been a food source in the region for more than a century; however, overfishing, pollution, and inadequate regulation have decreased populations, placing them at risk of extinction (Berlanga-Robles et al., 2002; Soto-Galera et al., 1998). In recent years, charal fishing has declined due to factors such as overexploitation and environmental degradation (Orbe-Mendoza et al., 2002), a situation that places not only the charales but also the residents of these regions in a vulnerable situation who regularly consume this resource and who are exposed to emerging contaminants such as microplastics. Therefore, in this study, we investigated the abundance, morphology, and distribution of microplastics in water, zooplankton, and fish from two shallow tropical water bodies in Central Mexico. This work will contribute to one of the first references on microplastic contamination in Mexico and could be helpful as a basis for monitoring these contaminants in tropical freshwater systems.

## Materials and methods

### Study area

**Fig. 1:**
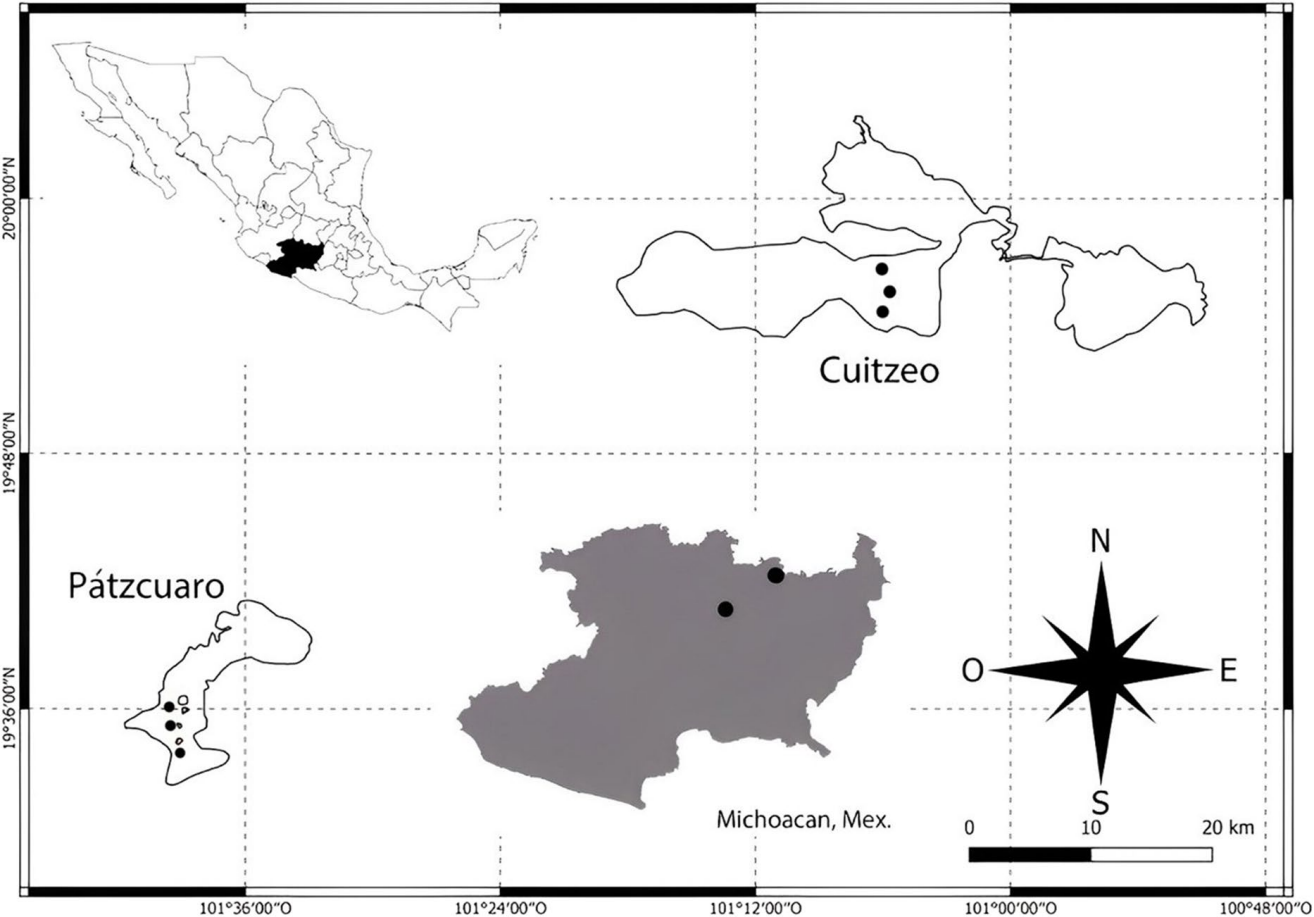
Sampling location map showing sites in Lakes Cuitzeo and Pátzcuaro, Michoacán State, Central Mexico

Lakes Cuitzeo and Pátzcuaro are located in the center of the country in the State of Michoacán, Mexico, between 20°05′–19°52′N and 100°50′–101°19′W and 19°32′–19°42′N and 101°32′–101°43′W respectively (Fig. 1). These water bodies are part of the Lerma-Chapala endorheic basin (Bernal-Brooks, 1998; Bradbury, 2000; Torres, 1993). They are shallow tropical lakes of high altitude since Cuitzeo is located at 1820 and Pátzcuaro at 2035 m a.s.l. (Bernal-Brooks, 1998). Lake Cuitzeo has a larger surface area of water bodies, with 420 km^2^, while Pátzcuaro has a maximum surface area of 116 km^2^; however, both lakes are considered among the largest in Mexico (Alcocer & Bernal Brooks, 2010). Both water bodies experience rain in summer (May to October) with an average annual precipitation of 906 mm (García Amaro, 2004; Bravo-Inclán et al., 2022). The trophic state of both lakes has varied over time between eutrophic and hypereutrophic, the latter being the prevailing one in aquatic systems (Berry et al., 2011; Lyons et al., 2000). In these lakes, there are five species of the genus *Chirostoma*, *C. estor*, *C. granduncle*, *C. patzcuaro*, *C. attenuatum*, and *C. humboldtianum*, commonly called charales, which have been historically consumed as part of the diet of the populations adjacent (Barbour, 1973; Berlanga-Robles et al., 2002; Lyons et al., 2000; Orbe-Mendoza et al., 2002). Currently, charal populations are subject to multiple stressors, mainly due to the eutrophication of water bodies, the introduction of exotic species, and the municipal and industrial waste dumped into them.

### Sample collection

The samples were collected in March 2020 in two tropical lakes in Central Mexico, Cuitzeo and Pátzcuaro. Three sampling sites were established in each lake, where triplicate samples of surface water (< 0.30 m) were collected using the bucket sampling method described by Song et al. (2014). For each sample, 30 L of water was filtered with a clean steel bucket through a prewashed stainless steel mesh with a 50-µm mesh size. The residue on the mesh was placed in prewashed glass containers with 100 mL of ultrapure water. The samples were preserved with 5% formalin and 4 °C until laboratory analysis. The fish collection was carried out by local fishermen, who used artisanal methods with traditional techniques that include seines, gillnets, butterfly nets, hooks, and harpoons (Orbe & Acevedo, 1991; Orbe-Mendoza et al., 2002) from whom we acquired the specimens immediately after being caught. Particularly in the collection of the specimens, gillnets were used in the places where the water samples were collected. In each lake, at least 150 fish of the genus *Chirostoma* were acquired, which were preserved in 10% formalin and then transferred to 70% alcohol and transferred to the laboratory for analysis.

### Laboratory analysis

In the laboratory, prior to analysis, 300 rotifers and 150 microcrustaceans (cladocerans and copepods) were randomly isolated from each of the nine water samples from each lake and processed independently. To remove organic matter from water samples and isolated zooplankton, we used the digestion technique with H_2_O_2_ in the presence of metal ion (Baldwin et al., 2016; Masura et al., 2015). We use hydrogen peroxide (30%) as an oxidant and an iron sulfate solution as a catalyst. To remove salts and minerals, we use density separation. Subsequently, the samples were vacuum filtered through a 0.22-µm pore size filter (GF/F Whatman). The filters were placed in clean Petri dishes for visual analysis under a microscope. A Nikon SMZ800 stereoscopic microscope (× 10– × 80) was used, where the microplastics were classified, counted, and isolated considering their color, size, and shape according to established categories (Hidalgo-Ruz et al., 2012). Based on their shape, microplastics were classified into three categories: fiber, fragment, and pellet (Rochman et al., 2019; Wang et al., 2017).

### Hydrogen peroxide for soft fabrics

The protocol of Li et al. (2016) was used to extract the particles from the gastrointestinal tracts of the fish. A total of 120 individuals from each lake were rinsed with ultrapure water, and biometrics (weight and size) were performed (Lusher et al., 2016), classifying them into four categories according to weight. The specimens were dissected by removing the gastrointestinal tract, from the esophagus to the anus, and ten gastrointestinal tracts were placed in a 500-mL beaker with 200 mL of H_2_O_2_ and incubated at 65 °C until the solutions appeared clear without particle evidence. Incubation times were 24 h and, in some cases, 48 h. For each of the four categories, three repetitions were carried out with ten gastrointestinal tracts each, so 120 fish were used (10 specimens × 3 repetitions × 4 categories).

### Identification and characterization

From the 60 filters obtained from the water, zooplankton, and fish samples, subsamples of microplastics were obtained that were used for the analysis of the micromorphological characteristics by scanning electron microscopy and for the verification of the polymer composition by Raman spectroscopy (Lusher et al., 2016). For SEM analyses, the microplastics were isolated and placed individually on carbon adhesive tape, coated with gold, and analyzed in a JEOL JSM6360LV microscope (SAMEB of ICMyL UNAM). Microplastics were identified using Raman spectroscopy at the University Laboratory of Spectroscopic Characterization, LUCE ICAT UNAM. Raman spectrum measurements were recorded with a WITec alpha300 RA (WITEc GmbH, Ulm, Germany) instrument using a 300 lines/mm grating and 532-nm laser light excitation, originating from a Nd:YVO4 green laser. The incident laser beam with a power of 44 mW was focused by × 20, × 50, and × 100 objectives (Zeiss, Germany) with 0.4, 0.75, and 0.9 NA, respectively. Punctual Raman spectra were obtained with 0.5-s integration time and ten accumulations. The data processing and analysis were performed with the WITec Project version 5.1 software.

### Quality assurance and quality controls

During all stages of the study (sampling and laboratory), measures were implemented to avoid potential contamination of the samples. Any plastic equipment in the sample collection was eliminated, and all field equipment used were carefully prewashed three times with ultrapure water. Cotton laboratory coats and clean nitrile gloves were used during sampling and laboratory work. Water samples at each sampling site were collected in triplicate and were covered, preserved, and stored immediately to avoid exposure to air. White controls were also carried out to verify potential contamination in the field and laboratory, following the same processes used in the samples. In the field, 5 L of ultrapure water was filtered, and after rinsing the 50-µm steel mesh, the water was placed in containers equal to those of the samples and the same analysis process of the environmental samples followed. This procedure allowed us to determine that possible contamination during sampling, transportation, and laboratory procedures was negligible. In addition to the white controls, in the laboratory, all glassware were washed using natural fiber cleaning brushes, rinsed three times with ultrapure water, and immediately covered with aluminum foil. The samples always remained covered during the laboratory processes to avoid airborne contamination. The observation of the filters under a stereoscopic microscope was carried out in clean Petri dishes. Prior to any laboratory analysis, an air purifier with a HEPA filter was used in the workspace.

### Statistical analysis

Normality tests were carried out with the Shapiro–Wilk test to compare the abundances of microplastics in water and zooplankton samples, while significant differences between groups were carried out with *t*-tests. If the normality test failed, a Mann–Whitney test was performed. After checking normality for comparisons between categories of fish, an analysis of variance (one-way ANOVA) was performed, followed by a Tukey multiple comparison. All these analyses were performed using the Prism software GraphPad 9.

## Results and discussion

### Abundance of microplastics in water, zooplankton, and fish

**Fig. 2:**
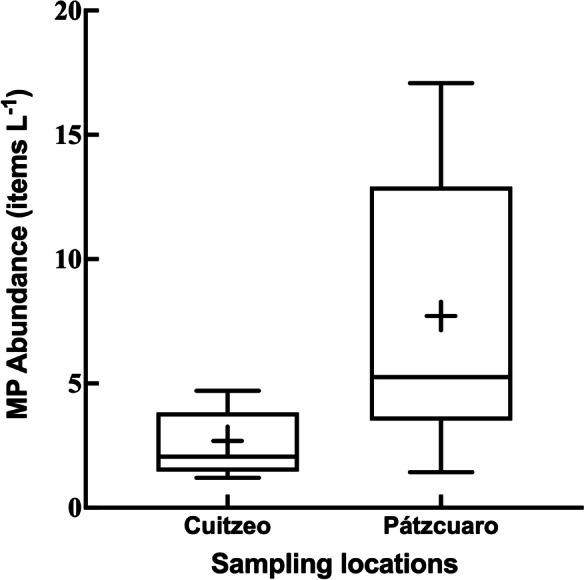
Box and whisker plots showing total microplastic densities in the water column in Lakes Cuitzeo and Pátzcuaro (for each box, the solid horizontal lines from top to bottom indicate maximum value, 75% quartile, median, 25% quartile, and minimum value, respectively; the symbol + inside the box represents the mean value)

Microplastics were widely detected in the surface water of the studied areas where average concentrations of 2.6 ± 0.4 items L^−1^ and 7.7 ± 1.8 items L^−1^ were recorded in Lakes Cuitzeo and Pátzcuaro respectively (Fig. 2). When comparing the total abundance of microplastics in surface water, significant differences were found (*p* = 0.01) between Lake Pátzcuaro, which presented values greater than 15 items L^−1^, and Lake Cuitzeo, where the maximum values were below 5 items L^−1^ (Fig. 2). The concentrations of microplastics detected in the surface waters of the lakes of Central Mexico were high considering the values obtained in other freshwater systems (Anderson et al., 2017; Fischer et al., 2016), especially from countries like China, which is the largest producer of plastics worldwide and where high concentrations of up to 8925 ± 1591 items/m^3^ MPs have been recorded (Jian et al., 2020; Wang et al., 2017, 2018). Elevated concentrations of microplastics in freshwater systems have been related to industrial and domestic waste (Browne et al., 2011), so proximity to urban centers impacts the abundance of microplastics (Barrows et al., 2018; Hendrickson et al., 2018; Wang et al., 2017). In the basins of these lakes, there are about 30 municipalities where 1 million people live. These municipalities, together with industrial activities, produce 700 tons of solid waste per day, half of which is plastics and industrial packaging (Delgado et al., 2007, 2008), which can potentially impact aquatic systems.

**Fig. 3:**
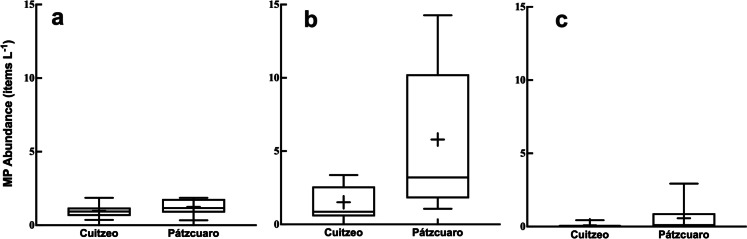
Box and whisker plots showing different microplastic forms: (**a**) fiber, (**b**) fragment, and (**c**) pellet densities in the water column in Lakes Cuitzeo and Pátzcuaro (for each box, the solid horizontal lines from top to bottom indicate maximum value, 75% quartile, median, 25% quartile, and minimum value, respectively; the symbol + inside the box represents the mean value)

Through the concentration analysis, the three forms of microplastics (fiber, fragments, and pellets) found in the study sites were higher. In the fraction corresponding to water, an average of 0.9 ± 0.14 items L^−1^ fibers was recorded in Lake Cuitzeo, while 1.2 ± 0.17 items L^−1^ fibers was found in Lake Pátzcuaro (Fig. 3a). Fragments were the most abundant forms in the surface water. They presented significant differences (*p* = 0.02) between both lakes with averages of 1.5 ± 0.39 and 5.7 ± 1.67 items L^−1^ fragments in Cuitzeo and Pátzcuaro respectively (Fig. 3b). Finally, in water, the forms with the lowest abundances were pellets with 0.08 ± 0.04 items L^−1^ in Lake Cuitzeo and 0.5 ± 0.3 items L^−1^ in Lake Pátzcuaro (Fig. 3c). The three forms of plastics, fibers, fragments, and pellets found in the surface waters of the lakes studied, are some of those that are typically recorded in epicontinental water bodies (Anderson et al., 2017; Jian et al., 2020; Wang et al., 2017, 2018; Xiong et al., 2021). However, our data differ in two main aspects from these works. The first is that in our study, the presence of films was not recorded, and the second is that the shape of the dominant particles was not fibers. Although films are forms that can be found in negligible concentrations (Xiong et al., 2021), they are forms frequently recorded in various continental water bodies (Zhao et al., 2022). On the other hand, fibers were the dominant particles in these works regarding the proportion of shapes, while in our study, the dominant shapes were fragments. In other studies, fragments are one of the most abundant forms in freshwater aquatic systems (reviewed by Koelmans et al., 2019). In Central Mexico, in a study where the presence and abundance of microplastics in sediments of the Atoyac River were analyzed (Shruti et al., 2019), it was found that the two dominant forms were films (25.9%) and fragments (22.2%). Fibers have a greater distribution in freshwater environments, while forms such as fragments and films have been mainly related to areas with anthropogenic influence (Amrutha & Warrier, 2020).

### Abundance of microplastics in zooplankton

**Fig. 4:**
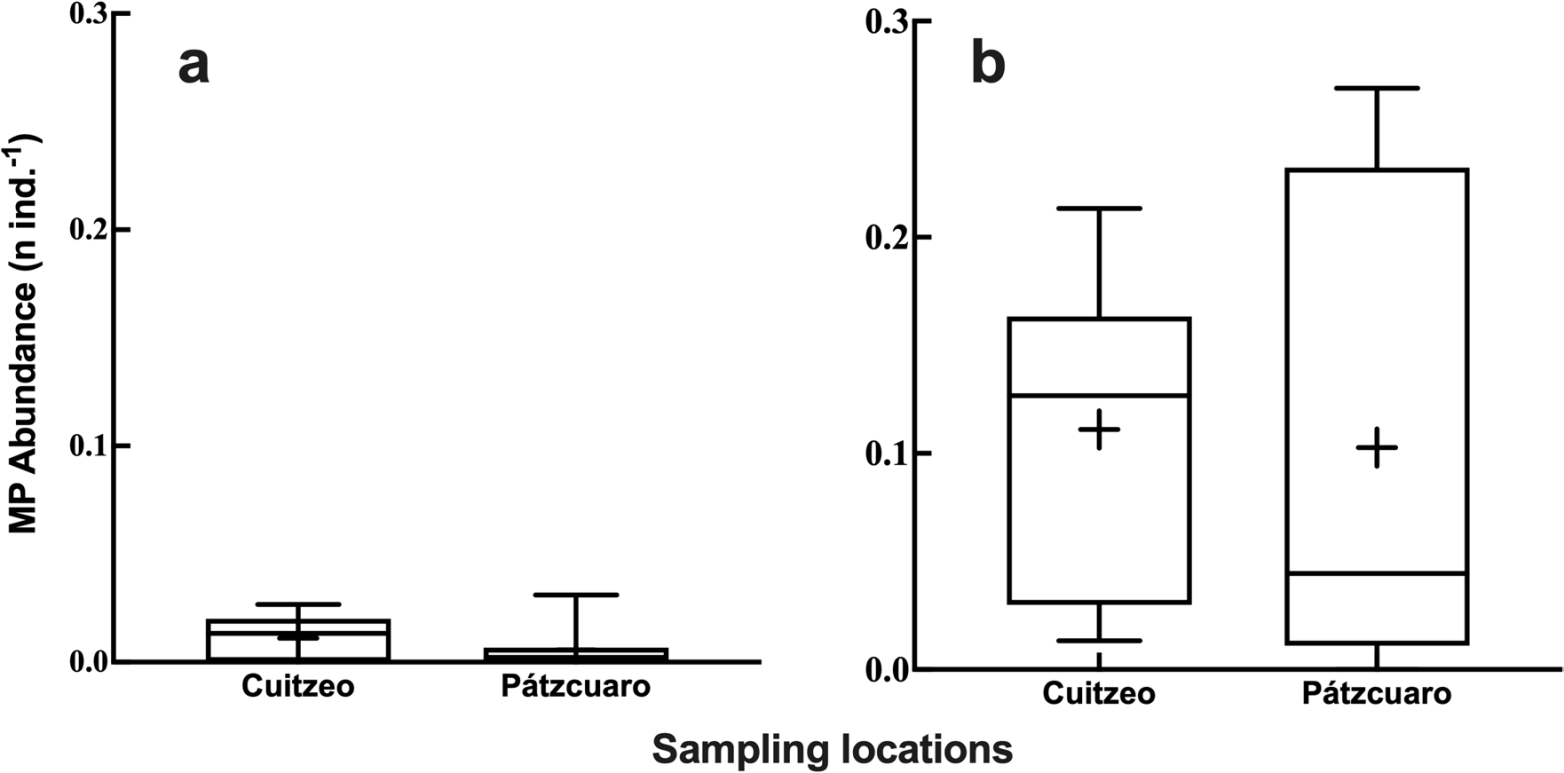
Box and whisker plots showing different microplastic forms: (**a**) fiber and (**b**) fragment densities in zooplankton collected in Lakes Cuitzeo and Pátzcuaro (for each box the solid horizontal lines from top to bottom indicate maximum value, 75% quartile, median, 25% quartile, and minimum value, respectively; the symbol + inside the box represents the mean value)

Of the 18 zooplankton samples analyzed in both lakes, the presence of microplastics was found in 94.5%. The abundance of microplastics in zooplankton had higher average values in Lake Cuitzeo in the two forms of microplastics observed in this group. Averages of 0.01 ± 0.003 items ind^−1^ fibers and 0.11 ± 0.02 items ind^−1^ fragments were recorded in Lake Cuitzeo, while Lake Pátzcuaro presented 0.005 ± 0.003 items ind^−1^ fibers and 0.10 ± 0.03 items ind^−1^ fragments (Fig. 4). No significant differences (*p* < 0.05) were observed within the zooplankton in the two forms of microplastics recorded in both lakes. Literature on microplastics in zooplankton still needs to be expanded. The initial efforts have been carried out mainly in marine environments and only correlate the concentrations of microplastics with zooplankton abundances (Moore et al., 2001; Lattin et al., 2004; Collignon et al., 2014; Frias et al., 2014). However, the study conducted by Sun et al. (2017) analyzed the ingestion of microplastics by zooplankton in the northern South China Sea. Finding that 100% of the zooplankton samples contained microplastics, so it was possible to verify that groups such as copepods in natural systems can ingest microplastics, as happened in our study where the presence of microplastics was recorded in 94% of the cases. In later works, such as the one carried out in the southern South China Sea (Amin et al., 2020), it was recorded that the consumption of microplastics by cyclopoids and calanoids copepods was between 0.003 and 0.13 particles ind^−1^, very similar values found in our work (Fig. 4). However, our study was carried out in a freshwater system including other zooplankton groups in addition to copepods such as rotifers and cladocerans.

Work with freshwater zooplankton has mainly been carried out in laboratory tests. It has been documented that zooplankton, particularly rotifers and microcrustaceans (cladocerans and copepods), are capable of consuming microplastics at rates between 2.5–3200 P ind^−1^ h^−1^ and 1–28,000 P ind^−1^ h^−1^, respectively (reviewed by Scherer et al., 2018). Laboratory studies have not only verified the consumption of microplastics by zooplankton, but they have also documented their adverse effects on the population dynamics of rotifers (Drago et al., 2020; Sun et al., 2019; Xue et al., 2021), cladocerans (De Felice et al., 2019), and copepods (Cole et al., 2013). According to our results, the three main groups of freshwater zooplankton (rotifers, cladocerans, and copepods) are capable of consuming microplastics under natural conditions. However, the effects observed in laboratory tests cannot be comparable with natural populations since higher concentrations of microplastics are used in laboratory tests (Phuong et al., 2016). The consumption of microplastics by zooplankton under natural conditions is a function of the abundance of zooplankton and the number of particles per individual, which is why it becomes inconsistent (Amin et al., 2020). The effect of microplastics on the food web using concentrations close to those recorded in natural water bodies was evaluated by Yıldız et al. (2022), who observed that zooplankton could easily ingest microplastics. However, the degree and incidence of ingestion were lower than those observed in laboratory tests. Likewise, their results show that the effect on the population dynamics of zooplankton due to the consumption of microplastics is less than that observed in laboratory tests.

### Abundance of microplastics in fish

**Fig. 5:**
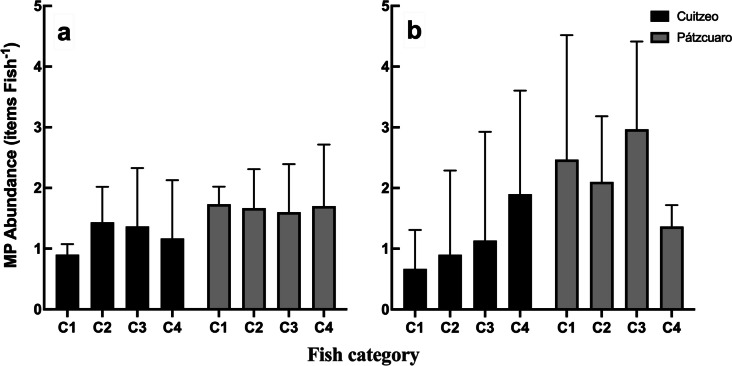
Microplastic densities: (**a**) fiber and (**b**) fragment from the gastrointestinal tract of *Chirostoma* fish collected in Lakes Cuitzeo and Pátzcuaro. Categories were established according to the fish weight: Lake Cuitzeo C1 0.5–0.7 g, C2 0.8–1.1 g, C3 1.2–1.4 g, and C4 1.5–2.1 g. Lake Pátzcuaro C1 2.4–2.8 g, C2 2.9–3.2 g, C3 3.3–3.6 g, and C4 3.7–4.3 g

The predominant form of microplastics in fish from both lakes was fragments, followed by filaments. No significant differences (*p* < 0.05) were observed within the fish categories in the two forms of microplastics recorded in this group of organisms. The range of particle abundance in the gastrointestinal tract of fish varied between 0.2 and 4.5 items ind^−1^ (Fig. 5). Only in the abundance of fragments in the fish from Lake Cuitzeo was a positive correlation between the weight of the organisms and the abundance of particles (Fig. 5b). Our results show that 100% of fish samples in both lakes consumed microplastics, which is consistent with published literature on microplastic consumption by freshwater fish (Campbell et al., 2017; Park et al., 2020a; Peters & Bratton, 2016; Phillips & Bonner, 2015), where variations between 8 and 100% have been recorded in the intake of microplastics. As in work carried out with different species of freshwater fish around the world (Silva-Cavalcanti et al., 2017; Park et al., 2020a; Wang et al., 2020; Garcia et al., 2021), in the fish collected in our survey study in water bodies of Central Mexico, the two predominant forms of microplastics in the gastrointestinal tract were fibers and fragments. The study carried out by Martínez-Tavera et al. (2021) with freshwater fish (*Oreochromis niloticus*) in a reservoir in central Mexico recorded that the fish predominantly had fibers in their gastrointestinal tract (up to 24 items sample^−1^), without recording any other form of microplastic.

The abundances recorded in *Chirostoma* fish in our study were lower than those recorded in the digestive tract of freshwater fish from South Korea, where average concentrations of 22.0 ± 14.6 particles fish^−1^ were recorded (Park et al., 2020a). However, our data fit more closely with those obtained in freshwater fish from Colombia (Garcia et al., 2021), Brazil (Silva-Cavalcanti et al., 2017), and the USA (Peters & Bratton, 2016) where averages of 2.1 ± 1.26 individual items^−1^, 3.6 items fish^−1^, and 1.65 items fish^−1^, respectively, were observed. Although the concentration of microplastics in both water bodies was high, this is not always reflected in fish’s high ingestion of microplastics. The ingestion of microplastics by fish is not necessarily related to the availability or abundance of microplastics in the water column; it is also related to the feeding habits of fish (McNeish et al., 2018). For example, fish capable of exploiting a wider variety of resources could ingest microplastics from the water column, mistaking them for prey or ingest them through contaminated prey (Scherer et al., 2018). In this case, the importance of the shape and color of microplastics becomes especially relevant since it has been shown that some species of fish preferentially consume some shapes and colors of microplastics, which may be related to the similarity to their natural prey regardless of its concentration in the water column (Okamoto et al., 2022; Ory et al., 2017; Scherer et al., 2018). In the case of the charal (*Chirostoma*) studied in our work, they are considered primarily zooplanktivorous and with the possibility of consuming benthic invertebrates (Berlanga-Robles et al., 2002; Orbe-Mendoza et al., 2002) so they would mainly be consuming microplastics from contaminated prey or by mistaking their prey for some microplastics. Finally, in different works, it has been suggested that the consumption of microplastics by fish is a route of transfer to humans (Erkes-Medrano et al., 2015; Akoueson et al., 2020; Garcia et al., 2021), in the case of *Chirostoma* fish perhaps more pronounced, because these fish are widely consumed in the center of the country and the entire organism is usually consumed (Orbe-Mendoza et al., 2002).

### Surface morphology, physical appearance, and elemental composition of microplastics

**Fig. 6:**
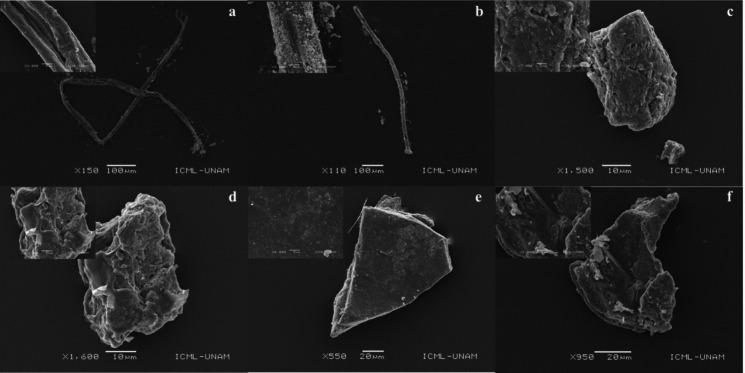
SEM images showing the degradation process on morphological features of selected microplastic forms from the three compartments: (**a**, **b**) fibers (water); (**c**, **d**) fragments (zooplankton); and (**e**, **f**) fragments (Fish)

**Fig. 7:**
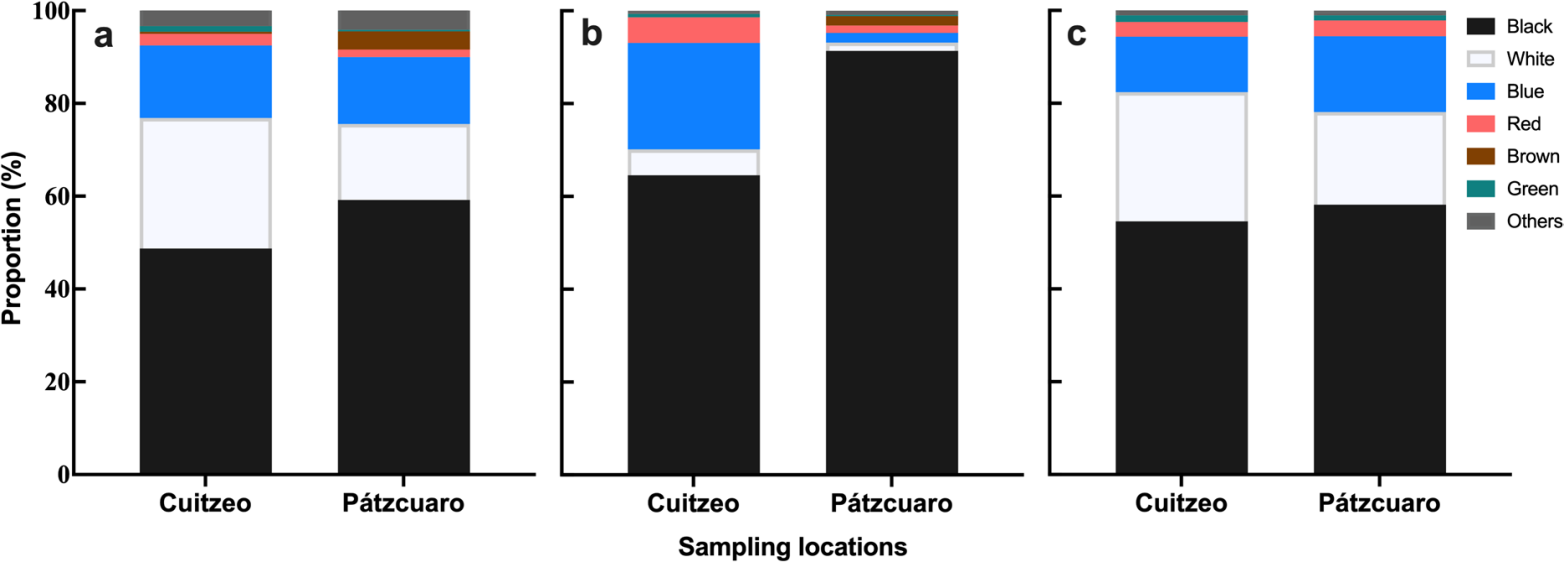
Distribution of microplastic color in three compartments: (**a**) water, (**b**) zooplankton, and (**c**) fish collected in Lakes Cuitzeo and Pátzcuaro

The surface of the particles analyzed by SEM showed various surface textures, among which grooves, fractures, mechanical pits, and scales stand out among others (Fig. 6). The color distribution of the microplastics in both bodies of water (Fig. 7) was black, white, blue, red, brown, and green; additionally, other colors such as purple, pink, transparent, yellow, and green were found in low percentages (< 1%). In the three fractions analyzed, water, zooplankton, and fish, the color with the highest percentage was black (> 50%), followed by white (< 28%) and blue (< 22%). In this work, 20 particles were analyzed with Raman spectroscopy to determine their chemical composition. The analysis showed that the main polymers found were polypropylene (PP), polyamide (nylon), and polyethylene terephthalate (PET). The surface textures of the particles analyzed in SEM, regardless of their shape, fibers, or fragments, clearly showed a degradation phenomenon. Coincidentally, in studies where secondary microplastics have been analyzed in freshwater bodies of water, the same signs of degradation have been observed in the particles such as fractures and mechanical pits, grooves, and scales (Jian et al., 2020; Garcia et al., 2021; Martinez-Tavera et al., 2021). Various factors are responsible for the erosion and degradation of plastics in the environment, including mechanical factors such as waves, oxidative weathering due to exposure to UV rays, and biological degradation (Zbyszewski & Corcoran, 2011, Zbyszewski et al., 2014). Analyzing the ultrastructure of microplastics not only can contribute to the historical reconstruction of the particles (Erkes-Medrano et al., 2015) but also gives us an idea about the effects it can have on its interactions with the biota since degradation signals can be related to more significant physical damage or increased toxicity in the organisms that consume them (Teuten et al., 2007; Wardrop et al., 2016). Coinciding with the results of our work, the color distribution of microplastics in various works around the world shows that colored plastics are dominant in water samples and ingested by organisms (De Sá et al., 2018; Eriksen et al., 2013; Wang et al., 2017, 2018).

Additionally, black color was the dominant color in the particles (> 50%) in our study, while in other works, the blue particles were the dominant ones (Güven et al., 2017; Peters & Bratton, 2016; Shruti et al., 2019; Lusher et al., 2020; Wu et al., 2018). In Mexico, Martinez-Tavera et al. (2021) recorded a dominance of black particles in fish collected in a reservoir, highlighting that this color of particles mainly comes from agricultural processes. Finally, the main polymers recorded in our work, polypropylene (PP), polyamide (nylon), and polyethylene terephthalate (PET), are the main polymers found in bodies of water, zooplankton, and freshwater fish (reviewed by De Sá et al., 2018; Koelmans et al., 2019; Parker et al., 2021; Zhao et al., 2022) and that have been related to urbanization and various human activities such as fishing in the case of polyamide (Barrows et al., 2018; Hossain et al., 2019; Peters & Braton, 2016; Shruti et al., 2019).

## Conclusions

This research is the first to provide data on the distribution of microplastics in three different strata of tropical water bodies in Mexico. The results show the presence of microplastics in the water column, zooplankton, and fish from both water bodies in the country’s center. The chemical composition of the microplastics was mainly polypropylene (PP), polyamide (nylon), and polyethylene terephthalate (PET). The dominance of the fragments in the three strata of the waterbody does not allow us to distinguish whether the presence of microplastics in the digestive tract of fish of the genus *Chirostoma* is carried out indirectly through the consumption of their prey (zooplankton) or directly from the water column, so future work is required to clarify the route of entry of these contaminants into fish.

## Data Availability

No datasets were generated or analysed during the current study.

## Declarations

All the authors have read and understood, and have complied as applicable with the statement on ethical responsibilities of authors as found in the Instructions for Authors.
